# Hospital service focus vs. breadth: Impact on hospital outcomes and the moderating role of hospital size

**DOI:** 10.1007/s10729-025-09741-7

**Published:** 2026-01-28

**Authors:** Matthew J. Castel, Timothy C. Dunne

**Affiliations:** 1 College of Business and Economics, Department of Information Technology and Supply Chain Management, Boise State University, 1910 University Drive, Boise, ID 83725-1615 USA; 2 College of Business and Economics, Department of Management, Boise State University, 1910 University Drive, Boise, ID 83725-1625 USA

**Keywords:** Hospital focus, Breadth of services, Hospital performance, Regression

## Abstract

There is an ever-increasing need for hospitals in the United States to improve upon their performance. In particular, it is necessary for hospitals to decrease their costs while improving patient satisfaction. Intuitively, hospitals adopt different strategies to accomplish those goals. Researchers have examined how hospitals that use a focus strategy (i.e., specialization) seek ways to improve performance by increased efficiencies and coordination among resources. Other studies examine the impact of increased hospital services (i.e. breadth) as a means to benefit from economies of scope. This study expands upon those literatures by submitting that focus and breadth do not have to be opposing strategies but can be implemented simultaneously; i.e. breadth of services with specialized focus on a few. The current study also examines how hospital size moderates the relationship between those two hospital strategies and performance. Specifically, this study applies an organizational information processing theory lens to predict that hospital focus and service breadth will impact patient satisfaction and cost per discharge, and how those relationships will be moderated by hospital size. Using a pooled cross-section, a regression analysis shows that hospital focus generally improves patient satisfaction while lowering cost; however, the impact on patient satisfaction is diminished for large hospitals. Additionally, service breadth tends to decrease patient satisfaction and lowers cost per discharge; however, the decrease in patient satisfaction is partially mitigated for large hospitals.

## Highlights

- Insights into how both hospital focus and service breadth can impact hospital performance.
- Leverage a unique viewpoint of focus as an emphasis of services.
- Focus and breadth both decrease cost per discharge, regardless of hospital size.
- Focus increases patient satisfaction for small hospitals, but its effect is diminished for large hospitals.
- Service breadth decreases patient satisfaction, but its effect is partially mitigated for large hospitals.

## Introduction

Skinner [1] introduced the idea of the focused factory, suggesting that operations which limit the scope of production to a specific product line achieve efficiencies that are not observable in factories that produce multiple product lines. These factory gains provide the ability for the factory to differentiate themselves from their competition and provide a cost advantage [2]. The idea that specialization (focus) provides the basis for improved performance and efficiency has led to multiple studies exploring this phenomenon in the manufacturing literature. Similarly, extensions of the focus concept have been extended into services, and in particular, healthcare. This is most evident within the famous case-study exploring Shouldice Hospital.

Shouldice Hospital – located in Ontario, Canada – demonstrated how focus, through the specialization of hernia procedures, allows for decreased costs and improved patient outcomes [3]. Shouldice has seen a 99.5% success rate over a 70-year period while additionally being 50% less expensive than general hospitals in the province of Ontario [4]. Similar benefits have been seen through clinics, like the Kensington Eye Institute, which was able to perform 7,200 cataract surgeries per year, at 23% lower cost and a reduction of wait times by nearly 60% [5]. However, such hospital and clinic specializations are extreme examples of focus, whereby they limit their area of care to a specific procedure or set of procedures – i.e., the diagnostic-resource group (DRG) level. In a similar vein, focus can also be seen as a specialization of major organ systems – i.e., the medical diagnostic category (MDC) level. While hospital specialization in cardiac and orthopedic procedures have become more common place, specialty hospitals make up a small proportion of hospitals in the United States.

Given that specialty hospitals are an exception to the rule, it becomes important to understand how focus is manifested in a “normal” hospital. Unlike manufacturing, which focuses on products, hospitals are a service-based industry. While Skinner’s [1] original definition of focus suggests a reduction in offerings, McLaughlin et al. [6] identified that service operations differ from their manufacturing counterparts. Within service environments, focus lends itself to identifying how the service organization addresses the “differentiation and selection of market segments and adjustment of the process and infrastructure parameters of the service delivery system to meet the needs of those specific market segments,” [6]. The definition of focus in a service organization is therefore the emphasis of a subset of services out of the entire service offering. For example, a hospital can have multiple service areas: cardiac, orthopedics, nephrology, etc. Focus would be emphasizing one area of care, such as cardiac care, over the other areas of care, while breadth would be the total offering of services. This aligns with McLaughlin et al. [6] who suggests that focus in the healthcare environment does not necessarily mean a reduction or narrowing of services (i.e. breadth), rather an emphasis on a given service line or set of service lines which allow for the service firm to provide value to their target customer segment. This definition was advanced in hospital focus research, with Clark and Huckman [7] identifying that focus in cardiovascular care leads to spillover effects into other areas of care. They suggest an emphasis of a specific clinical service can benefit from strong capabilities in complementary areas. While the literature has a large number of studies looking at hospital focus, there is a dearth of research that explores the parsed-out effects of hospital focus as an emphasis and the breadth of services offered at the hospital level (see 6.). Furthermore, the literature viewing focus as an emphasis is sparse limiting the understanding of how hospitals can use focus in a subset of services to improve efficiencies and patient satisfaction.

While certain specialty hospitals have a focus strategy described above, others offer a wide range of services, which often includes both inpatient and outpatient care, surgical services, diagnostics, specialized treatment, as well as other supporting services. The logic for this more full-service approach is that the organization would benefit from economies of scope by sharing resources across those services and providing effective care at an improved cost [8]. Thus, in addition to contributing to the literature that looks at the effect of focus as an emphasis, the current work seeks to simultaneously examine service breadth to determine how the two strategies impact hospital performance. In this regard, most studies assume focus is the opposite of service breadth. Therefore, a main contribution of our work is to show how focus and breadth can both be utilized to impact hospital performance. Given that focus can occur in hospitals that have a variety of service offerings brings about the research questions:

1. Will focus as an emphasis provide benefits to hospital outcomes?
2. Will service breadth of offerings provide benefits to hospital outcomes?

While hospital focus and service breadth are strategies that can be employed by hospitals to better meet patient needs and improve care, the size of a hospital is a structural limitation. Larger hospitals tend to have a greater number of resources (beds, nurses, equipment, etc.), but also have a more complex operating environments compared to small hospitals [9]. As the complexity increases, issues arise in providing care. While hospital cost and patient outcomes can be affected by the strategy the hospital employs, the literature has identified that hospital size may have an impact on outcomes when evaluating case-mix levels within the hospital [10]. However, we are unaware of any studies that examine hospital size as a moderator on the relationship between these strategies and performance. Thus, the current work will extend the growing literature on how hospitals can improve both patient satisfaction and cost. Accordingly, our third research question is as follows:

3) What effect does hospital size have on the effect of hospital strategies and outcomes?

In the remainder of the paper, we discuss the theoretical underpinnings of the study by describing organizational information processing theory (OIPT) and generate our hypotheses. This is then followed by a discussion of our data collection, analysis, and results. The paper concludes with a discussion and limitation of the study’s results.

## Literature review

### Organizational information processing theory (OIPT)

“Hospitals consist of multiple interdependent departments that frequently have conflicting goals and priorities but must coordinate their goals and priorities to deliver care,” [11]. Patients present with a heterogeneous set of conditions requiring a variety of treatments based upon age, condition, and comorbidities. This is further complicated by multiple providers having preferences for treatments and procedures. From an operations standpoint, this creates a high level of complexity and uncertainty that must be managed by the hospital and also requires coordination across departments to provide care.

Strategy research recognizes that effective planning and delivery of products and services is based upon the firm’s ability to effectively process information [12, 13]. Organizational information processing theory (OIPT) posits that organizational benefit derives from effective information processing, specifically by focusing on relevant data to reduce uncertainty in decision-making. The organization’s structure is meant to “coordinate the interdependent subtasks which result from the division of labor” [14]. With increasing uncertainty, the ability of the firm to process information becomes more difficult and the coordination of tasks is hampered. Information processing is defined as “the gathering, interpreting, and synthesis of information in the context of organizational decision making” [15]. The greater the amount of information processing required creates issues for the organization to synthesize and execute a plan of action creating what is called task uncertainty. For example, if a patient comes to a hospital complaining of chest pains, information related to that patient’s medical history, risk factors, medications, and other pertinent information are necessary to provide effective care. Thus, hospitals need a way to receive that information and to make sense of it as it relates to decisions made about how to care for the patient. Improving the organizational structure (e.g., implementing a hospital information system) to facilitate the effective processing and interpretation of information enables the hospital to provide superior quality services to patients. Galbraith [14] identified that this can be done in one of two ways: (1) reducing the information processing needs by limiting the task environment, or (2) increase the information processing capabilities of the firm.

**Fig. 1:**
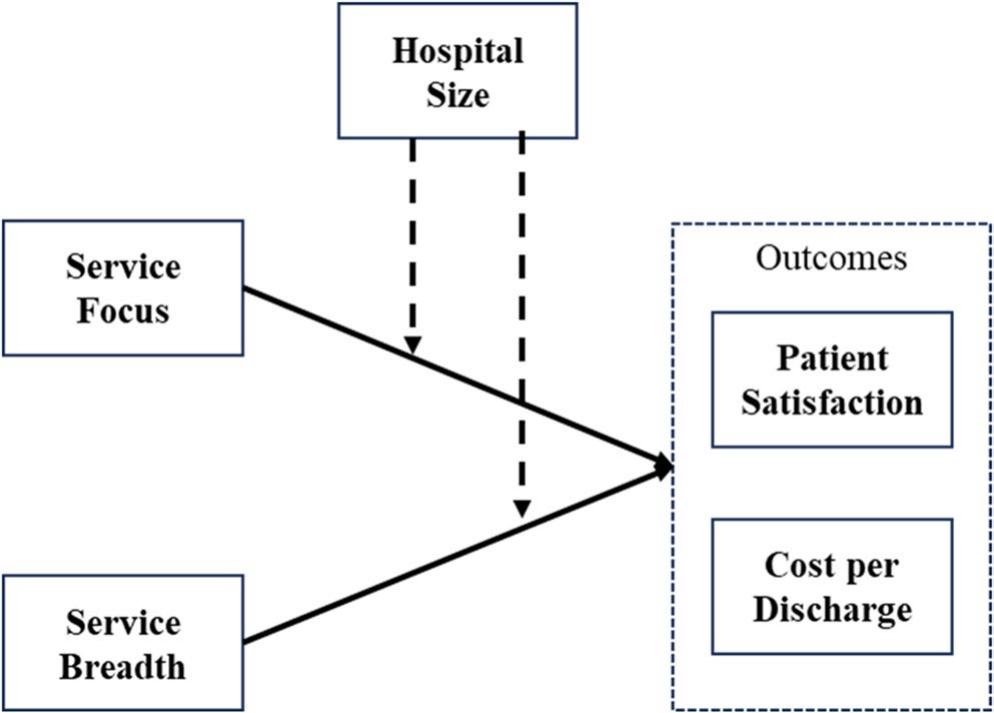
Theoretical model

Both recommendations by Galbraith [14] are based in the idea of having resources available for additional information processing. By reducing the information processing needs, the focal firm can create self-contained task environments and allow for efficiencies. In a hospital this can be achieved by creating departments responsible for a limited set of tasks or limiting the number of services offered – i.e., focus. However, even by creating self-contained task environments, like departments, requires coordination between the departments which can hinder efficiency. Similarly, by increasing the information processing of the firm, there must be resources available (e.g., people or IT systems) to process information and provide coordination across the task environment. Our theoretical model, developed further below, is represented in Fig. 1.

### Focus, breadth, and hospital performance

The concept of a focused factory [1] has been touted as a strategy that provides greater levels of efficiency and reduced production costs as firms gain from learning effects and economies of scale. This concept has been extended into the service arena, identifying that focus is related more towards an alignment to a specific type of customer [6]. Within hospital-based research, Clark and Huckman [7] identified that focus within a hospital environment is more of an “emphasis” and not necessarily a reduction in services. This implies that hospitals can have a large portfolio of services, but emphasize a few. With respect to focus, the healthcare research has demonstrated that focus can potentially improve service and process quality [7, 16–20], reduce mortality rates [16], lower the length of stay [16], and increase patient satisfaction [21]. Additionally, hospitals tend to see cost efficiencies associated with focus [8, 22]. OIPT implies that a focused set of services reduces uncertainty and coordination requirements, leading to improved outcomes. These efficiencies establish economies of scale related to focus. Subsequently, the excess capacity generated by these efficiencies can be leveraged by expanding the breadth of service offerings to attain economies of scope.

Traditionally, focus is viewed as the antithesis of breadth of services [23]. In light of hospital focus being an “emphasis” in specific service areas [7], there is the possibility of a hospital to have a full range of services and focus in a limited number of them. Breadth of services has been associated with the potential to have superior gains [24, 25] by making use of available resources and capabilities [26]. This typically leads to economies of scope allowing for multiple service lines to effectively use the resources of the firm. However, under OIPT, this suggests that there will be higher levels of coordination required within the firm. As a result, costs associated with additional services may be higher. While focus has demonstrated to benefit outcomes and avoid such coordination costs, breadth of services within a service area [27] and across complimentary service areas [7] allow for the hospital to provide for more complex conditions while improving patient acquisition and hospital outcomes, respectively. Recently, Parker-Lue and Lieberman [28] have shown that hospitals expanding from kidney transplants to liver transplants can see slightly improved performance – but at a cost. While there have been examples within specific MDCs or across DRGs, Clement [29] noted that linking service breadth to improved financial outcomes have not been clearly demonstrated; however, in their study, breadth was viewed as service diversification, not the extent of services provided.

In the case of focus and service breadth, there are also costs associated with providing care. Emphasizing on a specific set of conditions provides the firm the benefit from potential economies of scale. However, because resources may be available and underutilized, there is also the potential for breadth to leverage those resources allowing for better cost savings as well vis-à-vis economies of scope. Thus, we have the following hypotheses:

- H1a: *Hospital focus will negatively affect cost per discharge*.
- H1b: *Hospital service breadth will negatively affect cost per discharge*.

OIPT identifies that a reduction in the task environment complexity reduces the level of information processing and should enhance performance capabilities. Hospital focus limits the attention of the hospital to an emphasized set of service offerings and allows for ease of coordination. The resulting simplification of the care process reduces complexity, which directly facilitates enhanced patient care coordination. Because patients will be receiving care that is highly specialized and better coordinated for in this focused environment, they are expected to experience higher levels of satisfaction [e.g., 30]. Conversely, increasing service offerings raises hospital-patient coordination complexity and stretches resources across a wider domain. The resulting constraint on available resources limits the hospital’s ability to fully address patient needs. Based on this mechanism, we expect that heightened focus will lead to higher patient satisfaction, while an increase in service offerings will lead to lower patient satisfaction.

- H2a: *Hospital focus will positively affect patient satisfaction*.
- H2b: *Hospital service breadth will negatively affect patient satisfaction*.

### Effects of hospital size on information processing

In a seminal study evaluating the relationship between hospital size and cost, it was noted that while larger hospitals can more effectively utilize specialized personnel and equipment, they are simultaneously challenged by increase coordination issues (i.e., communication and control) [31]. The literature suggests that economies of scope, linked to the more efficient usage of resources, tend to realize greater cost reductions when there is a breadth of services [8, 31]. Similar studies in other countries, such as Ireland, have also noted that larger hospitals tend to run more efficiently than smaller hospitals [32].

While the complexity of coordinating care becomes more difficult as the size of a hospital increases, we posit that there are still the benefits of economies of scope. As larger hospitals treat more patients and generate greater revenue, they are often able to invest in more sophisticated technologies for improving efficiency [33] as well as the ability to train and develop staff more effectively [34]. Yet, we expect those benefits of economies of scope to be greater for hospitals with a focus strategy than those with a service breadth strategy. Studies find that some of the coordination and complexity challenges that come with size, are often offset by increased revenue that can overcome those issues for greater levels of profit [35] and efficiency [36]. We propose that this will be the case when hospitals have a focus strategy, where efficiencies can be gained due to the fact that those extra investments can be focused within fewer service areas. Conversely, we propose hospitals that offer a broad set of services, will not see that same benefit by increases in size. Thus, we hypothesize the following moderation hypotheses:

- H3a: *Hospital size will moderate the relationship between hospital focus and cost per discharge*,* such that the relationship will be stronger for larger hospitals.*
- H3b: *Hospital size will moderate the relationship between hospital service breadth and cost per discharge*,* such that the relationship will be weaker for larger hospitals.*

As an organization increases in size, the complexity of managing the task environment becomes more difficult. When viewing demographic and structural factors, in particular hospital beds, larger hospitals tend to have lower patient satisfaction [37] when compared to smaller hospitals [9, 37, 38]. McFarland et al. [37] found this evident with HCAHPS scores, with larger hospitals having lower scores in hospital cleanliness, reduced attentiveness of staff, and poor communication from doctors. Based upon OIPT, it is expected that as the size of the hospitals increase, the coordination of tasks becomes much more difficult and will have an impact on patient-based outcomes. This has the potential to diminish the effects of the strategies aimed at improving patient outcomes and expectations. Thus, we posit the following moderating relationship:

- H4a: *Hospital size will moderate the relationship between hospital focus and patient satisfaction*,* such that the positive relationship will be weaker for larger hospitals.*
- H4b: *Hospital size will moderate the relationship between hospital service breadth and patient satisfaction*,* such that the negative relationship will be weaker for larger hospitals.*

## Methods

### Data

The unit of analysis for the analysis was at the hospital level. The data from several sources were integrated to measure the independent variables, dependent variables, and control variables. The data were assembled from three databases: Nationwide Inpatient Sample (NIS), the Centers of Medicare and Medicaid Services (CMS) Final Rules files, and the Center of Medicare and Medicaid Services (CMS) Hospital Compare databases. This allowed for the data to cover 2008-2011 in a pooled, cross-sectional dataset. The NIS data provides a sample of hospitals and all their associated admissions, allowing for us to construct measures related to hospital focus and service breadth. However, given the structural changes to the NIS data starting in 2012, no later years of data could be utilized due to changes in the data collection.

### Hospital performance

*Cost per discharge* (*CPD*) was calculated similar to Senot et al. [39]. The charges to the patients were identified in the NIS data and averaged. This value was then multiplied by the hospital-specific inpatient cost-to-charge ratio, as provided by CMS. The natural log of CPD (*lnCPD*) was then taken to enable normally distributed residuals.

*Patient satisfaction* (*PatSat*) was measured using the HCAHPS survey in the CMS Hospital Compare database. Utilizing the ratings of the hospital, 0 = worst hospital possible to 10 = best hospital possible, patients are asked to rate their stay at the hospital. These answers are then presented by HCAHPS as percentages in the categories of “0–6”, “7–8”, and “9–10”. While some studies use only the top category [e.g., 40] of the HCAHPS survey items, we incorporate the negative and neutral responses utilizing a method similar to Chandrasekaran et al. [41]. The answer categories were coded “0–6” = −1, “7–8” = 0, and “9–10” = 1. These were then multiplied by their corresponding percentages and added together to get an overall patient satisfaction score. Thus, a more positive score reflects higher patient satisfaction, while a more negative score is indicative of lower patient satisfaction.

### Independent variables

Traditionally, focus and breadth are viewed as opposite sides of a continuum. Within manufacturing environments, focus is viewed as the production of a limited number of products, representing a restricted amount of variety. However, in service environments, focus is not necessarily the reduction of services, rather an emphasis within a broader portfolio of services [cf., 6,7]. While many authors have assessed focus [see 42] and breadth [e.g., 43,24], most of these measures account for the number of services (products) and the concentration of services (products) across the service categories. We attempt to break down these concepts in our measurements using ecology-based measures to assess the breadth of services and the unevenness (focus as an emphasis) of service usage. Doing this allows us to assess both the effects of service breadth and of focus as an emphasis.

*Service Breadth.* Consistent with using OIPT, we view service breadth as the extent of variety that exists within the task environment [cf. 44]. As such, service breadth (*breadth*) was simply measured by the number of service categories (*S*) the hospital provides. Given that hospitals typically hire or grant admitting rights based on specific organ systems, we quantified breadth by counting the number of unique MDCs observed through patient admissions.

1 $$Service\:Breadth=S_h$$

*Focus.* Hospital focus is often viewed by the literature as specialization [see 42]. Conceptualizing hospital focus as an emphasis on certain services suggests the application of the ecological measure of (un)evenness. Evenness measurements assess the degree to which a population is equitably distributed across the different categories within an environment [45]. Within a hospital environment, this would be the extent to which the patients presenting in a hospital are equitably spread across the categories of care (e.g., cardiology, obstetrics and gynecology, orthopedics, etc.). If there are concentrations within a subset of categories, it would demonstrate “unevenness” in the categories of care. Thus, hospital focus is measured as a lack of “evenness” using the ecological measure of Simpson’s evenness index [46] multiplied by a negative one. In the Eq. (2), *P* is the proportion of cases presenting in category of care (*i*) at hospital *h* across the total categories of care (*S*) present at the hospital. Similar to the argument for breadth, unevenness was measured at the MDC level.

2 $$\:Focus=\:-1*Evenness=\frac{-1}{{S}_{h}*\sum\:_{i=1}^{{S}_{h}}\left({P}_{ih}^{2}\right)}$$

*Hospital Size.* Hospital size (*Smll*, *Med*, *Lrg*) is measured utilizing the ordinal measures for bed size available in the NIS data. Hospital bed size was categorized by small (*Smll*), medium (*Med*), and large (*Lrg*)–- this is similar to what Lin et al. [47] did in their study of cost per discharge and hospital ownership. Hospital bed size was based upon the NIS definitions.

### Control variables

Given that this study utilized a pooled, cross-sectional dataset, we included year (*year*) dummy variables to control for time-based changes [48]. Hospital system membership (*member*), for profit status (*forprofit*), and teaching status (*teaching*) were included to control for structural limitations and requirements for the hospitals. Environmental influences resulting from location (*urban*) were also controlled for since urban environments tend to have greater access to resources. Finally, patient complexity was controlled for by incorporating the case mix index (*CMI*). CMI controls for patient comorbidities and has the potential to impact the type and cost of care provided.

## Results

### Descriptive results

The descriptive statistics and pairwise correlations are shown in Table 1.

**Table 1:** Descriptive statistics and pairwise correlations

		Mean	S.D.	[1]	[2]	[3]	[4]	[5]	[6]	[7]	[8]	[9]	[10]	[11]	[12]
[1]	Sys Member	0.52	0.50	1											
[2]	For Profit	0.16	0.37	0.15*	1										
[3]	Teaching	0.17	0.38	0.05*	-0.20*	1									
[4]	Urban	0.60	0.49	0.21*	0.16*	0.33*	1								
[5]	CMI	1.43	0.27	0.12*	-0.01	0.40*	0.39*	1							
[6]	Bed Size (Small)	0.46	0.50	-0.08*	0.05*	-0.15*	-0.08*	-0.16*	1						
[7]	Bed Size (Med)	0.24	0.43	0.02	-0.01	0.05*	0.06*	-0.11*	-0.52*	1					
[8]	Bed Size (Lrg)	0.30	0.46	0.06*	-0.05*	0.12*	0.03*	0.24*	-0.61*	-0.37*	1				
[9]	Focus	-0.40	0.12	0.08*	0.13*	-0.04*	0.16*	-0.03*	-0.04*	0.09*	-0.04*	1			
[10]	Breadth	21.60	4.05	0.08*	-0.18*	0.30*	0.19*	0.16*	-0.58*	0.23*	0.41*	0.09*	1		
[11]	Patient Satisfaction	55.90	13.86	0.003	-0.20*	-0.03	-0.15*	0.19*	0.25*	-0.10*	-0.14*	0.04	-0.30*	1	
[12]	ln(CPD)	9.01	0.42	0.09*	0.08*	0.24*	0.35*	0.64*	0.03	-0.05*	0.02	-0.001	-0.12*	0.14*	1

* *p* < 0.05

**Fig. 2:**
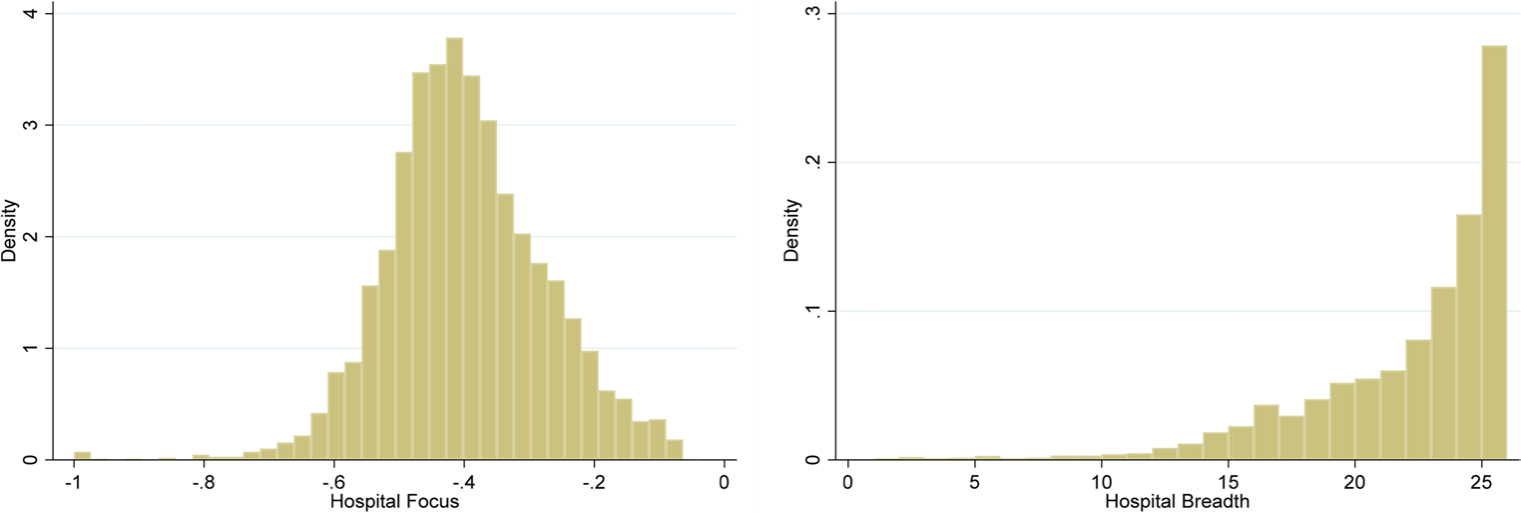
Histograms for focus and breadth

One thing to note in Table 2 is how *focus* and *breadth* appear to be separate strategy measures. While the two variables are correlated, the correlations and the direction of those correlations among other variables with those two strategies were highly variable. Looking at the histograms for *focus* and *breadth* in Fig. 2, *focus* tends to be fairly normal distributed, while *breadth* is more exponential in nature. This indicates more hospitals are likely to offer a larger number of services while low and high levels of focus are less common.

**Table 2:** Effects of hospital size, focus, and breadth on patient satisfaction and cost per discharge

	ln(Cost per Discharge)		Patient Satisfaction	
	Model 1	Model 2	Model 3	Model 4
Sys Member	0.005	0.006	1.556*	1.435*
	(0.013)	(0.013)	(0.664)	(0.653)
For Profit	−0.117***	−0.121***	−10.074***	−9.885***
	(0.019)	(0.019)	(1.040)	(1.031)
Teaching	0.047**	0.049**	−2.019*	−2.279**
	(0.018)	(0.017)	(0.816)	(0.772)
Urban	0.134***	0.136***	−4.918***	−5.830***
	(0.020)	(0.020)	(0.933)	(0.951)
CMI	0.166***	0.164***	4.392***	4.078***
	(0.010)	(0.011)	(0.466)	(0.459)
Med	−0.011	−0.004	−5.056***	−5.210***
	(0.020)	(0.019)	(0.936)	(0.914)
Lrg	−0.003	0.011	−5.876***	−6.972***
	(0.022)	(0.021)	(1.036)	(1.073)
Focus	−0.077***	−0.065***	0.429	2.121**
	(0.018)	(0.016)	(0.955)	(0.661)
Breadth	−0.032*	−0.038**	−3.313***	−2.754***
	(0.013)	(0.013)	(0.573)	(0.537)
Med x Focus		−0.010		−1.622^†^
		(0.019)		(0.907)
Lrg x Focus		−0.017		−1.890*
		(0.019)		(0.832)
Med x Breadth		0.023		1.344
		(0.022)		(0.918)
Lrg x Breadth		−0.010		2.802*
		(0.022)		(1.314)
Constant	8.916***	8.904***	56.594***	57.319***
	(0.038)	(0.037)	(1.556)	(1.491)
Year	Fixed	Fixed	Fixed	Fixed
Region	Fixed	Fixed	Fixed	Fixed
N	1458	1458	1647	1647
R^2^	0.549	0.550	0.238	0.251

^†^*p*<0.10, * *p*<0.05, ** *p*<0.01, *** *p*<0.001; Coefficients with robust standard errors; Default hospital bed size = small

Another interesting finding from the pairwise correlations is that *cost per discharge* is not correlated with *small* or *large* hospitals. Similarly, it is interesting that *focus* is not correlated with *patient satisfaction*.

### Regression results

Regression analysis using Stata 16 tested the hypotheses while controlling for the time-based, fixed effects resulting from the pooled, cross-sectional sample. The formulation for the regression models, where the dependent variables (DV) are patient satisfaction and cost per discharge, are shown in Eqs. 3 and 4 for the direct and interaction effects, respectively. Equation 3 tests hypothesis 1 (H1) in Model 1 and hypothesis 2 (H2) in Model 3. Equation 4 tests hypothesis 3 (H3) in Model 2 and hypothesis 4 (H4) in Model 4. The regression results for Models 1–4 are summarized in Table 2.

3 $$\begin{aligned} &\:DV={b}_{0}+{b}_{1}\left(focus\right)+{b}_{2}\left(breadth\right)+{b}_{3}\left(med\right)\\&+{b}_{4}\left(lrg\right)+{b}_{5}\left(member\right)+{b}_{4}\left(forprofit\right)\\ &+{b}_{5}\left(teaching\right)+{b}_{6}\left(urban\right)+{b}_{7}\left(cmi\right)+{b}_{8-11}\left(year\right)+\:\epsilon\:\end{aligned}$$

4 $$\begin{aligned}&\:DV={a}_{0}+{a}_{1}\left(focus\right)+{a}_{2}\left(breadth\right)+{a}_{3}\left(med\right)+{a}_{4}\left(lrg\right)\\&+{a}_{5}*\left(focus*med\right)+{a}_{6}*\left(focus*lrg\right)+{a}_{7}\left(breadth*med\right)\\&+{a}_{8}\left(breadth*lrg\right)+{a}_{9}*\left(member\right)+{a}_{10}\left(forprofit\right)\\&+{a}_{11}\left(teaching\right)+{a}_{12}\left(urban\right)+{a}_{13}\left(cmi\right)+{a}_{14-17}\left(year\right)+\:\epsilon\:\end{aligned}$$

Given the need to calculate interaction terms, the continuous variables on the right-hand side of the equations were standardized (e.g., *focus*,* breadth*,* CMI)* to ease interpretation. With cross-sectional studies, endogeneity can be of concern and several authors have suggested that hospital focus may be endogenous to its observed outcomes. This occurs because patients knowledgeable of a hospital that specializes in a set of procedures may show a preference for focused hospitals [27]. Furthermore, doctors may “cherry pick” the healthiest patients to get better outcomes and/or lower costs [16]. A similar argument could be made for service breadth, where patients with multiple ailments may go to a hospital with a greater number of services. This introduces the potential for endogeneity through omitted variable bias – i.e., an external variable influencing the independent and the dependent variable. We attempted to correct for endogenous effects by generating instrument variables for both *focus* and *breadth* using heteroskedasticity-based instrument variables (HBIV) [see 58]. HBIV attempts to generate instruments by leveraging the natural heteroskedasticity within the data to enable an instrument-free method of addressing endogeneity. Following the approach of Lewbel [49], the heteroskedasticity present in the error processes are used to generate the instruments. These instruments are then correlated with the endogenous variables allowing for identification of the model while controlling for potential endogeneity [49, 50]. The generated instrument variables were then used in the first stage of a two-stage least squares (2SLS) regression to limit the effects of endogeneity in the models described by Eqs. 3 and 4. The first-stage regression generated the estimated exogenous portion of the independent variables of interest (*IndVar*) – *focus* and *breadth* – with the *HBIVs* and the controls (*controls*); see Eq. 5.

5 $$\:\widehat{\left(IndVar\right)}={\alpha\:}_{0}+\boldsymbol{H}\boldsymbol{B}\boldsymbol{I}{\boldsymbol{V}}_{\boldsymbol{m}}\boldsymbol{*}{\alpha\:}_{m}+\boldsymbol{c}\boldsymbol{o}\boldsymbol{n}\boldsymbol{t}\boldsymbol{r}\boldsymbol{o}\boldsymbol{l}{\boldsymbol{s}}_{\boldsymbol{n}}\boldsymbol{*}{\beta\:}_{n}$$

The second-stage regression utilized the estimated values of $\:\widehat{focus}$ and $\:\widehat{breadth}$ obtained in the first stage to estimate Eq. 3. A similar procedure was used for Eq. 4. The entirety of the 2SLS regressions were estimated using the Stata command “ivreg2h” [49, 51].

The results of the 2SLS regressions testing the hypotheses (H1- H4) are shown in Table 2. Hypothesis 1 (Eq. 3, Model 1) posited that hospital focus will negatively affect cost per discharge (H1a) and that hospital service line breadth will negatively affect cost per discharge (H1b). The regression results in Model 1 show that the effect of hospital focus is negatively related to cost per discharge (b= −0.077, *p* < 0.001), and that hospital breadth is negatively related to cost per discharge (b= −0.032, *p* < 0.05), providing full support for H1a and H1b.

Hypothesis 2 (Eq. 3, Model 3) posited that hospital focus will positively affect patient satisfaction (H2a), but that hospital breadth will negatively affect patient satisfaction (H2b). The regression results in found partial support for H2. Specifically, the analysis found that hospital focus is positively related to patient satisfaction ($\:b$ = 0.429, *p* > 0.05), but the relationship is not statistically significant. Hospital breadth, however, is negatively related to patient satisfaction ($\:b$ = -3.313, *p* < 0.001), thus providing support for H2b but not H2a.

Hypothesis 3 (Eq. 4, Model 2) posited that the size of the hospital will moderate the relationships between hospital focus and cost per discharge, and between hospital breadth and cost per discharge. These hypotheses posited that the relationship between hospital focus and cost per discharge will be lower for larger hospitals (H3a), and the relationship between hospital breadth and cost per discharge will be weaker for larger hospitals (H3b). Reviewing the results of Model 2, it is noted that there was no difference between small, medium, and large hospitals with the *med* and *lrg* coefficients being insignificant. Additionally, H3a was not supported, with the Model 2 producing no significant interaction of large hospitals and hospital focus on cost per discharge ($\:\mathrm{b}$ = -0.017, *p* > 0.05). Also, H3b was not supported, with the interaction of large hospital and hospital breadth on cost per discharge ($\:\mathrm{b}$ = -0.010, *p* > 0.05) being not significant. This indicates the effect of hospital size does not have a significant effect on *CPD* and also shows a lack of a moderating impact for focus or breadth on *CPD*.

**Fig. 3:**
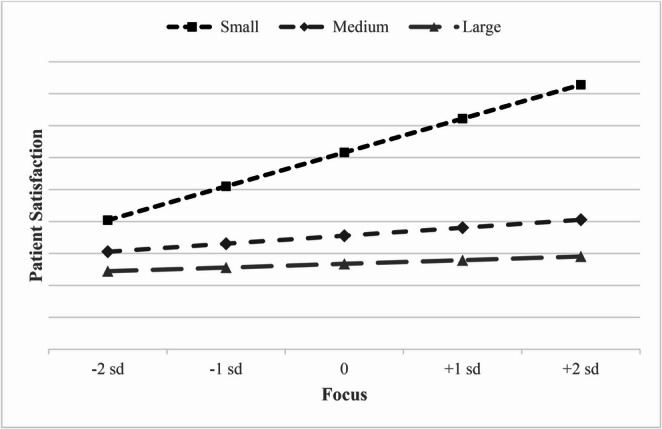
Bed size moderating the relationship between focus and patient satisfaction

**Fig. 4:**
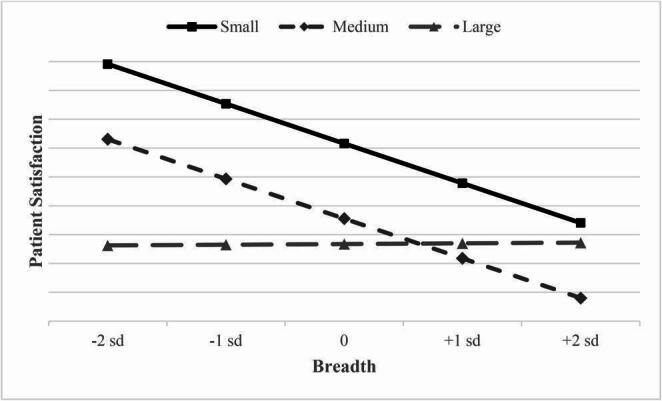
Bed size moderating the relationship between breadth and patient satisfaction

Hypothesis 4 (Eq. 4, Model 4) posited that the size of the hospital will moderate the relationships between hospital focus and patient satisfaction, and between hospital breadth and patient satisfaction. Specifically, we predicted that the relationship between focus and patient satisfaction would be weaker for large hospitals (H4a), and that the relationship between hospital breadth and patient satisfaction would also be weaker for large hospitals (H4b). As shown in Model 4, H4a is supported with the model indicating the interaction of large hospitals and hospital focus negatively affects patient satisfaction ($\:\mathrm{b}$ = -1.890, *p* < 0.05). However, the interaction of large hospitals and breadth on patient satisfaction was positive ($\:\mathrm{b}$ = 2.802, *p* < 0.05), which was contrary to our predicted direction; hence, H4b was not fully supported as hypothesized. Given the mixed results, we graphed the interactions of hospital size for *focus* and *breadth* on *patient satisfaction* in Figs. 3 and 4, respectively. Figure 3 shows that smaller hospitals have a noticeably higher slope than medium and large hospitals. This shows that smaller hospitals are associated with a larger benefit of focus on patient satisfaction than their medium and large hospital counterparts. In Fig. 4, breadth of services is shown to temper the negative effects a larger hospital has with patient satisfaction, while small and medium hospitals show a decline in patient satisfaction as the breadth of services increase.

### Robustness Checks

All of the models had regression diagnostics performed. Robust standard errors were used to prevent issues related to heteroskedasticity and outliers were removed to prevent bias in the estimators. None of the standard errors were unusually large, indicating the multicollinearity was not of concern [48]. The residuals of all the regressions appeared to be normally distributed. Omitted variable bias was limited through the usage of Lewbel’s [49] HBIV method and sample selection bias was reduced by the NIS data being representative of the population of U.S. hospitals in its data collection.

When a hospital only has one or two service lines (i.e. one or two MDCs), there is the predicament where unevenness will be equal to negative one (i.e. perfectly even). Additionally, this is the first instance we have seen unevenness measures used to assess hospital focus. To assess if this was a problem, we evaluated the models by varying minimum MDC-levels accepted within the sample. There were no significant differences in the effect sizes or significance levels when restricting for MDC counts to be greater than three, five, or eight. While the focus coefficient changed slightly, the direction and significance of the coefficients were similar.

Finally, measuring service breadth at the MDC level is indicative of assessing service-line breadth. However, MDCs are limited to 25 categories. Given that this may not fully represent the breadth of the services a hospital provides, we additionally assessed breadth at the DRG level. There are roughly 740 DRG codes representing diagnoses that patients present with. We utilized the primary DRG code patients were admitted for in lieu of the MDC code to assess the extent of breadth. This would be more representative of expanding actual service offerings instead of the service lines. As shown in Table 3, the magnitude of the service breadth coefficient changed, but the direction and significance of the variables were comparable to our original analysis.

**Table 3:** Effects of hospital size, focus, and breadth (DRG-level) on patient satisfaction and cost per discharge

	ln(Cost per Discharge)		Patient Satisfaction	
	Model 5	Model 6	Model 7	Model 8
Sys Member	0.008	0.006	1.902**	1.362*
	(0.013)	(0.013)	(0.697)	(0.650)
For Profit	−0.128***	−0.122***	−11.166***	−9.427***
	(0.021)	(0.019)	(1.094)	(1.028)
Teaching	0.082***	0.063***	1.267	−1.853*
	(0.024)	(0.019)	(1.167)	(0.846)
Urban	0.201***	0.149***	−0.131	−6.535***
	(0.036)	(0.025)	(1.635)	(1.356)
CMI	0.190***	0.175***	6.450***	4.514***
	(0.015)	(0.012)	(0.687)	(0.464)
Med	0.024	−0.026	−2.237	−7.000***
	(0.028)	(0.024)	(1.338)	(1.258)
Lrg	0.065	0.013	−0.13	−10.447***
	(0.042)	(0.032)	(1.982)	(1.863)
Focus	−0.099***	−0.069***	0.462	2.224***
	(0.019)	(0.017)	(1.017)	(0.667)
Breadth	−0.114**	−0.094***	−9.447***	−5.485***
	(0.043)	(0.028)	(2.052)	(1.163)
Med x Focus		−0.006		−1.683
		(0.019)		(0.912)
Lrg x Focus		−0.015		−1.807*
		(0.019)		(0.844)
Med x Breadth		0.072*		4.229**
		(0.029)		(1.484)
Lrg x Breadth		0.04		6.348***
		(0.030)		(1.590)
Constant	8.893***	8.928***	55.445***	60.297***
	(0.042)	(0.039)	(1.757)	(1.667)
Year	Fixed	Fixed	Fixed	Fixed
Region	Fixed	Fixed	Fixed	Fixed
N	1458	1458	1647	1647
R^2^	0.541	0.549	0.189	0.250

^†^*p*<0.10, * *p*<0.05, ** *p*<0.01, *** *p*<0.001; Coefficients with robust standard errors; Default hospital bed size = small

## Discussion

This study contributes to the literature in several ways. First, this study attempts to extend the literature in exploring focus as an emphasis. Expanding upon the works of Clark and Huckman [7] and Parker-Lue and Lieberman [28], this study goes beyond an individual organ system and procedures to explore the impact of focus and breadth agnostic of specialty area. Second, the study explores how hospital size impacts the outcomes of focus and breadth by introducing hospital size as a moderator.

Based upon the analysis, hospital focus– measured as the emphasis in a set of services –lowers cost per discharge; while breadth tends to decrease both patient satisfaction and cost per discharge. In terms of lowering cost per discharge, the current study supports the benefits of the economies of scale hospitals develop through focus, as well as the benefits of economies of scope from service breadth. These findings suggest that, all else being equal, service focus and service breadth may allow a hospital to decrease costs. When looking at the outcome of patient satisfaction, the effects of focus and breadth are not as equally beneficial. We found that patient satisfaction increases for small hospitals when hospitals focus on specific services, however, the effects are drastically diminished for larger hospitals. Furthermore, our results show that service breadth results in decreased patient satisfaction absent the interaction of hospital size. However, while larger hospitals generally have lower patient satisfaction than smaller hospitals, they have the potential to mitigate the decreased patient satisfaction associated with increasing service-line breadth. These findings suggest that patients greatly value the care provided when a hospital uses a focus strategy in small or medium-sized hospitals. However, the opposite effect occurs when these hospitals increase service breadth.

This research provides a major contribution, through the lens of organizational information processing theory (OIPT), to differentiate and simultaneously examine hospital focus and service breadth strategies, and to predict the moderating role of hospital size. Whereas prior studies have generally treated these concepts as mutually opposing strategies [e.g., 22, 27], applying OIPT allows us to assess distinctly how each strategy impacts a hospital’s inherent need for, and capacity to process, information.

Our findings extend the established OIPT literature by demonstrating that the strategic benefits of focus and breadth may be contingent upon organizational scale when looking at patient satisfaction. The result that hospital focus yields its greatest patient satisfaction benefit for small hospitals strongly supports OIPT’s core premise: constraining the scope of information processing effectively reduces environmental uncertainty and coordination demands [7], which provides a significant advantage when organizational complexity is low. Conversely, the negative direct effect of service breadth on patient satisfaction in smaller hospitals is consistent with OIPT’s prediction that increasing information processing and coordination leads to processing capacity overload. However, the moderation results for large hospitals present a counter-intuitive finding: the negative effect of breadth is mitigated by the interaction with hospital size.

This theoretical lens provides a critical viewpoint in potentially explaining the observed moderation effects. The finding that the negative effect of breadth is mitigated by large size suggests an alternative OIPT mechanism: the high volume associated with large hospitals enables routinization and standardization across service lines. This operational response minimizes the residual uncertainty and information processing needed for each patient encounter, thereby explaining the mitigation. This perspective also complements the view that large hospitals, through investments in advanced technology and specialized staff [33, 34], may enable them to effectively mitigate the negative coordination effects associated with service breadth. This robust structural capacity could explain why these large hospitals can maintain patient satisfaction levels despite increasing service complexity. However, this enhanced organizational complexity [31] simultaneously explains the significant attenuation of the positive focus effect in large hospitals; the sheer volume of inherent information and coordination challenges associated with scale overpower the targeted gains achieved through focus. This illustrates a unique structural trade-off when applying OIPT principles across varying organizational sizes.

## Limitations

Although this study provides a valuable contribution to the literature, several limitations should be acknowledged. First, the study relies on cross-sectional data, which limits the ability to make causal inferences. While the associations identified between hospital strategy, hospital size, and performance, are valuable to advance the field, it would benefit from a longitudinal study. Second, while we are looking at the moderating effects of bed size on the effects of hospital focus and service breadth, we do not directly address the mechanisms associated with *how* focus and breadth impact CPD and patient satisfaction. Future studies looking at resource slack, such as nurse utilization or physician to bed ratios, might provide additional insight into the mechanism. Furthermore, the measure of hospital bed size was limited due to the data. Evaluating bed size, or other comparable size variables, that are continuous may provide a more comprehensive view of how hospital size impacts outcomes and also yield a more nuanced insight. Despite these limitations, this study contributes to the growing literature and enhances what we know about the effects of hospital strategy and outcomes.

## Conclusion

Our study makes two major contributions to the literature. First drawing on the OIPT, we differentiate focus as an emphasis from service breadth to evaluate the effect of economies of scale and scope on cost per discharge and patient satisfaction. Second, our study explores the ramifications of hospital size as a moderator for the relationships between focus as an emphasis and service breadth on cost per discharge and patient satisfaction.

We identify that both hospital focus and service breadth are associated with reductions in cost per discharge across all hospital sizes. However, the influence on patient satisfaction is highly conditional, with the positive effect of focus being attenuated in larger hospitals. Conversely, the negative direct effect of service breadth is mitigated in large hospitals, meaning these facilities can maintain their patient satisfaction despite an increase in service breadth. These findings underscore the necessity for managers to tailor strategies based on existing scale when deciding whether to prioritize focus or service line expansion.

## Data Availability

The data used in the research are a combination of for-fee (licensed) and free data sources. These data are available through the following: • HCUP data is available to trained and authorized users for purchase through the Agency for Healthcare Research and Quality (AHRQ): https://hcup-us.ahrq.gov/tech_assist/centdist.jsp. • Patient satisfaction data collected by the Hospital Consumer Assessment of Healthcare Providers and Systems (HCAHPS) and is available through the Centers for Medicare and Medicaid Services (CMS) data archive: https://data.cms.gov/provider-data/archived-data/hospitals.

## Appendix A

**Table 4:** List of relevant studies exploring focus and breadth of hospital services

Author(s)	Journal	Independent Variable(s)	Moderator Variable(s)	Performance Measure	Findings/Summary
Carey et al. [52]	HSR	Specialization; Full-service	na	Cost	Hospitals specializing in orthopedic/surgical were less efficient than full-service hospitals; other specialties were no different in cost.
Hyer et al. [53]	JOM	Focus (unit level)	na	Length of stay; Mortality; Cost/Pay per patient	Cost associated with decreased length of stay and improvement in cost/pay per patient
KC and Terwiesch [16]	MS	Focus at firm level, unit level, and process flow level.	na	Length of stay; Mortality	Focus had different impacts on length of stay and mortality depending on the level of analysis.
McDermott and Stock [54]	JOM	Focus (as emphasis)	na	Cost	Found some associations between cost (depending on how it was operationalized) and cost.
Clark and Huckman [7]	MS	Focus (cardiac); Breadth (related diversification)	na	Mortality	Focus has a direct relationship on mortality; Breadth does not have a direct relationship, but interacts with focus to have a positive effect.
Andritsos and Tang [55]	POMS	Focus (cardiac)	na	Length of stay	Higher resource usage from a focused strategy was associated with a decrease in length of stay.
Ding [17]	JOM	Focus at department and hospital level; Experience	na	Efficiency	Focus was directly associated with efficiency; the interaction of focus and experience was associated with increased efficiency.
Ding [56]	JOM	Focus at department and hospital level	na	Performance in treating heart attacks	Focus was generally associated with reductions in mortality rates and readmittance due to heart attack procedures.
Ding et al. [22]	JOM	Service mix as specialization and differentiation	na	Cost	Increases in hospital specialization result is decreases in cost improvements; Differentiation improves cost.
Peng et al. [57]	DS	Elect. Medical records	Focus; Complexity	Process of care	EMR mitigates hospital complexity for improved process care. Focus moderates the relationship between EMR and process care.
Sfekas [27]	MSOM	Focus; Diversification	na	Consumer Choice	Customers prefer focus over diversification when selecting a hospital (obstetrics)
Freeman et al. [8]	MS	Focus (scale) Other (scope)	na	Cost	Focus was positively related to reduction of cost per patient.
Linde and Beilfuss [58]	JAMA	Hospital affiliations	na	Breadth; Cost	Clinicians working across multiple hospitals provide more service breadth and have higher costs
Ding [59]	JOM	Focus	na	Performance progression rates	The relationship between focus and performance is curvilinear.
Puro et al. [60]	HSR	Hospital characteristics (profit/non)	na	Breadth; Depth (focus)	Nonprofit hospitals provided greater breadth of services and greater depth than private hospitals.

Journal abbreviations as follows: *Decision Sciences* (DS); *Health Services Research* (HSR); *Journal of Operations Management* (JOM); *Journal of the American Medical Association* (JAMA); *Management Science* (MS); *Production and Operations Management* (POMS)
